# Grade 3/4 Adverse Event Costs of Immuno-oncology Combination Therapies for Previously Untreated Advanced Renal Cell Carcinoma

**DOI:** 10.1093/oncolo/oyac186

**Published:** 2023-01-18

**Authors:** Bradley McGregor, Daniel M Geynisman, Mauricio Burotto, Camillo Porta, Cristina Suarez, Maria T Bourlon, Viviana Del Tejo, Ella X Du, Xiaoran Yang, Selvam R Sendhil, Keith A Betts, Stephen Huo

**Affiliations:** Dana-Farber Cancer Institute, Boston, MA, USA; Fox Chase Cancer Center, Philadelphia, PA, USA; Bradford Hill Clinical Research Center, Santiago, Chile; University of Bari “A. Moro,” and Azienda Ospedaliero-Universitaria Consorziale Policlinico di Bari, Bari, Italy; Vall d´Hebron Institute of Oncology (VHIO), Hospital Universitari Vall d´Hebron, Vall d´Hebron Barcelona Hospital Campus, Barcelona, Spain; Instituto Nacional de Ciencias Médicas y Nutrición Salvador Zubirán, Mexico City, Mexico; Bristol Myers Squibb, Princeton, NJ, USA; Analysis Group, Inc., Los Angeles, CA, USA; Analysis Group, Inc., Los Angeles, CA, USA; Analysis Group, Inc., Los Angeles, CA, USA; Analysis Group, Inc., Los Angeles, CA, USA; Bristol Myers Squibb, Princeton, NJ, USA

**Keywords:** advanced renal cell carcinoma, adverse event cost, nivolumab plus ipilimumab, nivolumab plus cabozantinib, pembrolizumab plus axitinib, pembrolizumab plus lenvatinib

## Abstract

**Background:**

Despite 4 approved combination regimens in the first-line setting for advanced renal cell carcinoma (aRCC), adverse event (AE) costs data are lacking.

**Materials and Methods:**

A descriptive analysis on 2 AE cost comparisons was conducted using patient-level data for the nivolumab-based therapies and published data for the pembrolizumab-based therapies. First, grade 3/4 AE costs were compared between nivolumab + ipilimumab vs. nivolumab + cabozantinib vs. pembrolizumab + axitinib using data from the CheckMate 214 (median follow-up [mFU]: 13.1 months), CheckMate 9ER (mFU: 12.8 months), and KEYNOTE-426 (mFU: 12.8 months) trials, respectively. Second, grade 3/4 AE costs were compared between nivolumab + ipilimumab vs. nivolumab + cabozantinib vs. pembrolizumab + lenvatinib using data from the CheckMate 214 (mFU: 26.7 months), CheckMate 9ER (mFU: 23.5 months), and KEYNOTE-581 (mFU: 26.6 months) trials, respectively. Per-patient costs for all-cause and treatment-related grade 3/4 AEs with corresponding any-grade AE rates ≥ 20% were calculated based on the Healthcare Cost and Utilization Project database and inflated to 2020 US dollars.

**Results:**

Per-patient all-cause grade 3/4 AE costs for nivolumab + ipilimumab vs. nivolumab + cabozantinib vs. pembrolizumab + axitinib were $2703 vs. $4508 vs. $5772, and treatment-related grade 3/4 AE costs were $741 vs. $2722 vs. $4440 over ~12.8 months of FU. For nivolumab + ipilimumab vs. nivolumab + cabozantinib vs. pembrolizumab + lenvatinib, per-patient all-cause grade 3/4 AE costs were $3120 vs. $5800 vs. $9285, while treatment-related grade 3/4 AE costs were $863 vs. $3162 vs. $5030 over ~26.6 months of FU.

**Conclusion:**

Patients with aRCC treated with first-line nivolumab-based therapies had lower grade 3/4 all-cause and treatment-related AE costs than pembrolizumab-based therapies, suggesting a more favorable cost-benefit profile.

## Teaser

The efficacy and safety of novel immunotherapy-based combinations have been evaluated, but there is currently a paucity of evidence on the economic benefits and risks associated with these therapies. This information can be valuable to healthcare decision-makers and payers. This article compares descriptive analyses of all-cause and treatment-related grade 3/4 adverse events costs of nivolumab plus ipilimumab, nivolumab plus cabozantinib, pembrolizumab plus axitinib, and pembrolizumab plus lenvatinib as first-line treatments for patients with advanced renal cell carcinoma.

**Implications for Practice**

There is currently limited evidence on the economic benefits and risks associated with novel immunotherapy-based combinations used to treat patients newly diagnosed with advanced or metastatic renal cell carcinoma (aRCC). The present study addresses this gap in literature by comparing all-cause and treatment-related grade 3/4 adverse event costs of nivolumab-based and pembrolizumab-based combinations as first-line treatments for patients with aRCC using data from relevant clinical trials. The study results suggest that nivolumab-based combinations have a more favorable cost-benefit profile and offer clinicians and payers a therapeutic option that may reduce the substantial clinical and economic impacts for previously untreated patients with aRCC.

## Introduction

Renal cell carcinoma (RCC) is the most common type of kidney cancer, estimated to develop in more than 76,000 new patients and lead to nearly 14,000 deaths in the US in 2021.^1,2^ At diagnosis, approximately 25%-35% of patients have advanced or metastatic RCC (aRCC) which, until recently, was associated with a 5-year survival rate below 15%.^1,3,4^ Targeted therapies, such as the tyrosine kinase inhibitor (TKI) sunitinib, had been the standard of care for patients with previously untreated aRCC until recently.^5,6^ In the past 5 years, novel therapeutic options, including immunotherapy-based combination therapies, emerged for this population offering better disease control and improved survival outcomes.^7-10^

In April 2018, nivolumab plus ipilimumab was the first immunotherapy-based combination to be approved by the FDA for the first-line treatment of intermediate/poor-risk aRCC based on results of the CheckMate 214 phase III clinical trial (NCT02231749).^11^ Among all treated patients, 65% of those who received nivolumab plus ipilimumab had an all-cause grade 3/4 adverse event (AE) compared with 76% of the patients treated with sunitinib.^12^ Pembrolizumab plus axitinib was approved in April 2019 for the first-line treatment of patients with aRCC based on evidence from the KEYNOTE-426 trial (NCT02853331).^13^ In this trial, all-cause grade 3 or higher AEs occurred in 76% of patients in the pembrolizumab plus axitinib arm and 71% of patients in the sunitinib arm.^9^

The combination of nivolumab plus cabozantinib was granted FDA approval in January 2021 for treatment-naïve patients with aRCC in any-risk group based on the results of the CheckMate 9ER trial (NCT03141177)^14^ wherein all-cause grade 3 or higher AEs occurred in 75% of patients in the nivolumab plus cabozantinib arm and 71% of patients in the sunitinib arm.^8^ In August 2021, pembrolizumab plus lenvatinib was approved for the same indication based on the results of the KEYNOTE-581 trial, also known as the CLEAR trial (NCT02811861).^15^ Compared with sunitinib, treatment with pembrolizumab plus lenvatinib was associated with 82% vs. 72% all-cause grade 3 or higher AEs.^10^

While the efficacy and safety of these novel immunotherapy-based combinations has been evaluated, there is currently a paucity of evidence on the economic benefits and risks associated with these therapies. Given the marked impact of financial toxicity on patients with cancer in the US,^16^ this information can be valuable to healthcare decision-makers and payers. Therefore, the objective of this study was to compare descriptive analyses of all-cause and treatment-related grade 3/4 AE costs of nivolumab plus ipilimumab, nivolumab plus cabozantinib, pembrolizumab plus axitinib, and pembrolizumab plus lenvatinib as first-line treatments for patients with aRCC, using individual patient-level data (IPD) from the CheckMate 214 and CheckMate 9ER trials, as well as published data from the KEYNOTE-426 and KEYNOTE-581 trials.

## Materials and Methods

### Data Sources

Due to the different follow-up times from the KEYNOTE-426 and KEYNOTE-581 (CLEAR) trials for the adverse events reported in the KEYTRUDA prescribing information (median follow-up: 13.1 and 26.6 months, respectively),^9,10^ 2 distinct comparisons were conducted in order to ensure comparability in the follow-up time across the CheckMate and KEYNOTE trials. For the comparison of nivolumab plus ipilimumab (*N* = 547) vs. nivolumab plus cabozantinib (*N* = 320) vs. pembrolizumab plus axitinib (*N* = 429), IPD from the CheckMate 214 trial (data cutoff: August 31, 2016; median follow-up: 13.1 months) and the CheckMate 9ER trial (data cutoff: November 30, 2019; median follow-up, 12.8 months) for all treated patients were used to assess all-cause and treatment-related grade 3/4 AE rates associated with nivolumab plus ipilimumab and nivolumab plus cabozantinib, respectively. To obtain the all-cause and treatment-related grade 3/4 AE rates associated with pembrolizumab plus axitinib for all treated patients, published results of the KEYNOTE-426 trial (data cutoff: August 24, 2018; median follow-up: 12.8 months) from the KEYTRUDA prescribing information^17^ and Rini et al^9^ were used.

Similarly, for the comparison of nivolumab plus ipilimumab (*N* = 547) vs. nivolumab plus cabozantinib (*N* = 320) vs. pembrolizumab plus lenvatinib (*N* = 355), IPD from the CheckMate 214 trial (data cutoff: November 30, 2017; median follow-up: 26.7 months) and the CheckMate 9ER trial (data cutoff: September 10, 2020; median follow-up: 23.5 months) for all treated patients were used to obtain the all-cause and treatment-related grade 3/4 AE rates associated with nivolumab plus ipilimumab and nivolumab plus cabozantinib, respectively. For pembrolizumab plus lenvatinib, published results of the KEYNOTE-581 trial (data cutoff: August 28, 2020; median follow-up: 26.6 months) from the KEYTRUDA prescribing information^17^ and Motzer et al^10^ were used to obtain the all-cause and treatment-related grade 3/4 AE rates, respectively, for all treated patients.

Unit costs for grade 3/4 AEs were obtained from the US 2017 Healthcare Cost and Utilization Project (HCUP) National Inpatient Database, which are estimated from the US payer perspective. The HCUP database is a family of healthcare databases with a national information resource of encounter-level healthcare data (HCUP Partners) which is developed through a Federal-State-Industry partnership and sponsored by the Agency for Healthcare Research and Quality (AHRQ).^18-20^ As patients with grade 3/4 AEs require hospitalization based on the definitions by Common Terminology Criteria for Adverse Events (CTCAE), the national level of inpatient costs from the HCUP database were used to estimate the unit costs associated with each grade 3/4 AE. All costs were inflated to 2020 US dollars (USD) using an inflation factor of 1.0793 from 2017 USD based on the Consumer Price Index (CPI) for all urban consumers in medical care service.^21^

### Study Design

The analyses focused on all treated patients with clear cell aRCC, which represented the study population for all 4 clinical trials. All-cause and treatment-related grade 3/4 AEs with corresponding any grade AEs that occurred in at least 20% of patients in the nivolumab plus ipilimumab arm (CheckMate 214), nivolumab plus cabozantinib arm (CheckMate 9ER), pembrolizumab plus axitinib arm (KEYNOTE-426), and pembrolizumab plus lenvatinib arm (KEYNOTE-581) were considered in the cost calculation. The analysis assumed that each all-cause and treatment-related grade 3/4 AE occurred only once per patient treated in each arm of each trial since the KEYTRUDA prescribing information,^17^ Rini et al^9^ (for pembrolizumab plus axitinib), and Motzer et al^10^ (for pembrolizumab plus lenvatinib) only reported the percentage of patients experiencing each AE.

To be consistent with the reporting criteria of the KEYNOTE-426 trial, the analysis comparing nivolumab plus ipilimumab vs. nivolumab plus cabozantinib vs. pembrolizumab plus axitinib using IPD from CheckMate 214 and Checkmate 9ER included all AEs that occurred while patients received treatment and within 30 days after the end of the trial treatment period or within 90 days after the end of the trial treatment period for serious AEs. Likewise, to be consistent with the reporting criteria of the KEYNOTE-581 trial, the analysis comparing nivolumab plus ipilimumab vs. nivolumab plus cabozantinib vs. pembrolizumab plus lenvatinib using IPD from Checkmate 214 and Checkmate 9ER considered all AEs (including serious AEs) that occurred while patients received treatment or within 30 days after the end of the trial treatment period. To ensure a fair comparison, all-cause grade 3/4 AEs from the IPD of the CheckMate 214 and CheckMate 9ER trials were recategorized according to the definitions the KEYTRUDA prescribing information (Supplementary Tables S1 and S2).^17^

### Statistical Analysis

The cost associated with each grade 3/4 AE was calculated by multiplying the AE rate by the respective unit AE cost. The total per-patient costs of all-cause and treatment-related grade 3/4 AEs were then calculated by summing the costs of all relevant AEs and were descriptively reported for each treatment arm. The top cost drivers of each treatment arm were described. All analyses were performed using SAS 9.4 (SAS Institute, Inc., 100 SAS Campus Drive, Cary, NC 27513, USA).

## Results

### Nivolumab Plus Ipilimumab Vs. Nivolumab Plus Cabozantinib Vs. Pembrolizumab Plus Axitinib (Median Follow-Up of ~12.8 Months)

#### All-Cause Grade 3/4 AE Costs

**Figure 1.**
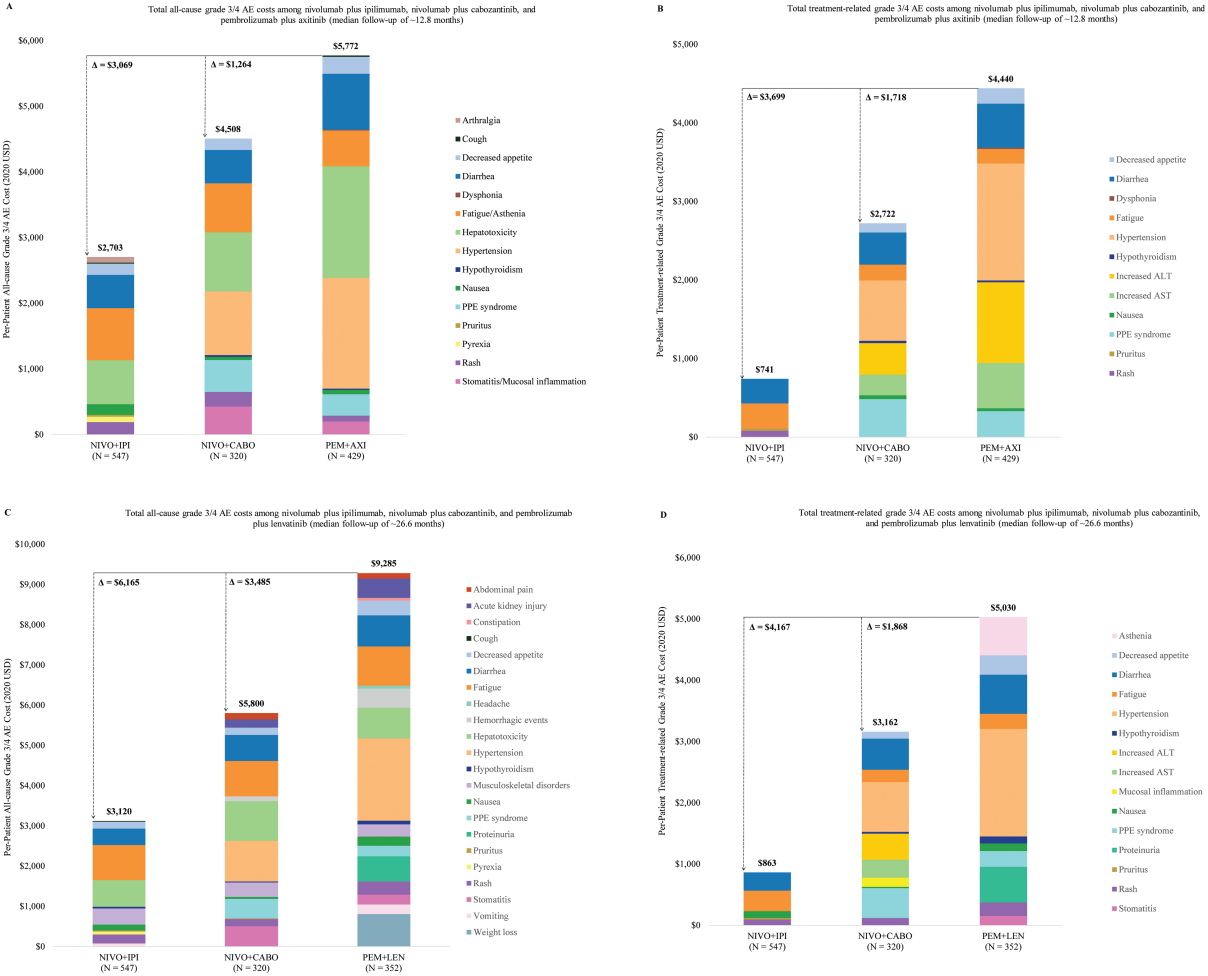
Total all-cause and treatment-related grade 3/4 AE costs^a^. Abbreviations: *AE: adverse event; ALT: alanine aminotransferase; AST: aspartate aminotransferase; NIVO + CABO: nivolumab plus cabozantinib; NIVO + IPI: nivolumab plus ipilimumab; PEM + AXI: pembrolizumab plus axitinib; PEM + LEN: pembrolizumab plus lenvatinib; PPE: palmar-plantar erythrodysesthesia; USD: United States dollar.* Note: [a] Grade 3/4 AEs with a corresponding any grade AE rate <20% were not included in the cost assessment and are not included in the figures.

The average all-cause grade 3/4 AE costs for patients with aRCC who received nivolumab plus ipilimumab vs. nivolumab plus cabozantinib vs. pembrolizumab plus axitinib are presented in Fig. 1A and Table 1. Patients treated with pembrolizumab plus axitinib incurred the highest all-cause grade 3/4 AE costs per patient ($5772). Those who received nivolumab plus cabozantinib incurred $4508 per patient (22% lower than pembrolizumab plus axitinib), while patients who received nivolumab plus ipilimumab incurred $2703 per patient (53% lower than pembrolizumab plus axitinib).

**Table 1.** All-cause grade 3/4 AE costs associated with nivolumab plus ipilimumab, nivolumab plus cabozantinib, and pembrolizumab plus axitinib (median follow-up of ~12.8 months).^a^

AE	Unit cost^b^	NIVO + IPI (*N* = 547)	NIVO + CABO (*N* = 320)	PEM + AXI (*N* = 429)
Arthralgia	$6513	$83	—	—
Constipation	$6687	—	—	$0
Cough	$8880	$16	—	$18
Decreased appetite	$9260	$169	$174	$259
Diarrhea	$7736	$509	$508	$851
Dysgeusia	$7471	—	$0	—
Dysphonia	$6819	—	—	$14
Fatigue/asthenia	$10 829	$792	$745	$541
Hepatotoxicity	$8514	$669	$905	$1703
Hypertension	$7023	—	$966	$1685
Hypothyroidism	$9673	—	$30	$19
Nausea	$7547	$166	$47	$68
PPE syndrome	$6480	—	$486	$324
Pruritus	$4690	$26	—	—
Pyrexia	$7608	$83	—	—
Rash	$6480	$190	$223	$91
Stomatitis/mucosal inflammation	$12 399	—	$426	$198
Total cost^c^		$2703	$4508	$5772

Notes: [a] “—” represents an AE that was not experienced in any grade by ≥ 20% of patients treated with nivolumab plus ipilimumab in the CheckMate 214 trial, nor among patients treated with nivolumab plus cabozantinib in the CheckMate 9ER trial or not reported among patients treated with pembrolizumab plus axitinib in the KEYNOTE-426 trial per the KEYTRUDA prescribing information. [b] AE unit costs were based on AE-related hospitalization cost, which were estimated from the Healthcare Cost and Utilization Project (HCUP) 2017 National Inpatient Database based on the ICD-10 diagnosis codes associated with each AE. Cost inputs were inflated from 2017 USD to 2020 USD using an inflation factor of 1.0793 based on the Consumer Price Index (CPI) for all urban consumers in medical care service. [c] Total costs were calculated by multiplying each AE rate by the corresponding AE unit cost and reflect the rounded sum of the individual AE costs in each treatment arm.

Abbreviations: AE, adverse event; NIVO + CABO, nivolumab plus cabozantinib; NIVO + IPI, nivolumab plus ipilimumab; PEM + AXI, pembrolizumab plus axitinib; PPE, palmar-plantar erythrodysesthesia.

In each treatment arm, the top 5 AEs with the highest costs contributed to the majority of the total all-cause grade 3/4 AE cost. Hepatotoxicity, hypertension, diarrhea, fatigue/asthenia, and palmar-plantar erythrodysesthesia (PPE) syndrome accounted for 88% of the total all-cause grade 3/4 AE cost related to pembrolizumab plus axitinib. In the nivolumab plus cabozantinib arm, the same AEs had the highest all-cause grade 3/4 AE costs and accounted for 80% of the total cost. For nivolumab plus ipilimumab, the top 5 AE cost drivers were fatigue/asthenia, hepatoxicity, diarrhea, rash, and decreased appetite, which accounted for 86% of the total all-cause grade 3/4 AE cost (Table 1).

#### Treatment-Related Grade 3/4 AE Costs

The average treatment-related grade 3/4 AE costs for aRCC patients who received nivolumab plus ipilimumab vs. nivolumab plus cabozantinib vs. pembrolizumab plus axitinib are presented in Fig. 1B and Table 2. Patients treated with pembrolizumab plus axitinib incurred the highest treatment-related grade 3/4 AE costs per patient ($4440). Those who received nivolumab plus cabozantinib incurred $2722 in treatment-related grade 3/4 AE costs per patient (39% lower than pembrolizumab plus axitinib), while patients who received nivolumab plus ipilimumab incurred $741 in treatment-related grade 3/4 AE costs per patient (83% lower than pembrolizumab plus axitinib).

**Table 2.** Treatment-related grade 3/4 AE costs associated with nivolumab plus ipilimumab, nivolumab plus cabozantinib, and pembrolizumab plus axitinib (median follow-up of ~12.8 months).^a^

AE	Unit cost^b^	NIVO + IPI (*N* = 547)	NIVO + CABO (*N* = 320)	PEM + AXI (*N* = 429)
Decreased appetite	$9260	—	$116	$194
Diarrhea	$7736	$311	$411	$559
Dysgeusia	$7471	—	$0	—
Dysphonia	$6819	—	—	$16
Fatigue	$7979	$321	$199	$186
Hypertension	$7023	—	$768	$1490
Hypothyroidism	$9673	—	$30	$23
Increased ALT	$8504	—	$399	$1031
Increased AST	$8504	—	$266	$575
Nausea	$7547	—	$47	$35
PPE syndrome	$6480	—	$486	$332
Pruritus	$4690	$26	—	—
Rash	$6480	$83	—	—
Total cost^c^		$741	$2722	$4440

Notes: [a] “—” represents an AE that was not experienced in any grade by  ≥ 20% of patients treated with nivolumab plus ipilimumab in the CheckMate 214 trial, nivolumab plus cabozantinib in the CheckMate 9ER trial or pembrolizumab plus axitinib in the KEYNOTE-426 trial, or not reported among patients treated with pembrolizumab plus axitinib in the KEYNOTE-426 trial, Rini et al. [b] AE unit costs were based on AE-related hospitalization cost, which were estimated from the Healthcare Cost and Utilization Project (HCUP) 2017 National Inpatient Database based on the ICD-10 diagnosis codes associated with each AE. Cost inputs were inflated from 2017 USD to 2020 USD using an inflation factor of 1.0793 based on the Consumer Price Index (CPI) for all urban consumers in medical care service. [c] Total costs were calculated by multiplying each AE rate by the corresponding AE unit cost and reflect the rounded sum of the individual AE costs in each treatment arm.

Abbreviations: AE, adverse event; ALT, alanine aminotransferase; AST, aspartate aminotransferase; NIVO + CABO, nivolumab plus cabozantinib; NIVO + IPI, nivolumab plus ipilimumab; PEM + AXI, pembrolizumab plus axitinib; PPE, palmar-plantar erythrodysesthesia.

In each treatment arm, the top AEs with the highest costs contributed to a majority of the total treatment-related grade 3/4 AE cost. Hypertension, increased alanine aminotransferase (ALT), increased aspartate aminotransferase (AST), diarrhea, and PPE syndrome accounted for 90% of the total treatment-related grade 3/4 AE cost associated with pembrolizumab plus axitinib. In the nivolumab plus cabozantinib arm, the same AEs had the highest treatment-related grade 3/4 AE costs and accounted for 86% of the total cost. For nivolumab plus ipilimumab, fatigue, diarrhea, rash, and pruritus contributed to 100% of the total treatment-related grade 3/4 AE cost (Table 2).

### Nivolumab Plus Ipilimumab Vs. Nivolumab Plus Cabozantinib Vs. Pembrolizumab Plus Lenvatinib (Median Follow-Up of ~26.6 Months)

#### All-Cause Grade 3/4 AE Costs

The average all-cause grade 3/4 AE costs for patients with aRCC who received nivolumab plus ipilimumab vs. nivolumab plus cabozantinib vs. pembrolizumab plus lenvatinib are presented in Fig. 1C and Table 3. Patients treated with pembrolizumab plus lenvatinib incurred $9285 in all-cause grade 3/4 AE costs per patient. Those who received nivolumab plus cabozantinib incurred $5800 per patient (38% lower than pembrolizumab plus lenvatinib), while patients who received nivolumab plus ipilimumab incurred $3120 per patient (66% lower than pembrolizumab plus lenvatinib).

**Table 3.** All-cause grade 3/4 AE costs associated nivolumab plus ipilimumab, nivolumab plus cabozantinib, and pembrolizumab plus lenvatinib (median follow-up of ~26.6 months).^a^

AE	Unit cost^b^	NIVO + IPI (*N* = 547)	NIVO + CABO (*N* = 320)	PEM + LEN (*N* = 352)
Abdominal pain	$6926	—	$152	$139
Acute kidney injury	$9573	—	$209	$479
Constipation	$6687	—	—	$67
Cough	$8880	$16	—	—
Decreased appetite	$9260	$169	$174	$370
Diarrhea	$7736	$410	$653	$774
Dysgeusia	$7471	—	$0	—
Dysphonia	$6819	—	—	$0
Fatigue	$10 829	$871	$880	$975
Headache	$7471	—	—	$75
Hemorrhagic events	$9462	—	$118	$473
Hepatotoxicity	$8514	$669	$984	$766
Hypertension	$7023	—	$1009	$2037
Hypothyroidism	$9673	$35	$30	$97
Musculoskeletal disorders	$7601	$403	$356	$304
Nausea	$7547	$152	$47	$226
PPE syndrome	$6480	—	$486	$259
Proteinuria	$7860	—	—	$629
Pruritus	$4690	$26	$15	—
Pyrexia	$7608	$70	—	—
Rash	$6480	$225	$182	$324
Stomatitis	$12 399	—	$504	$248
Vomiting	$8041	$73	—	$241
Weight loss	$10 044	—	—	$804
Total cost^c^		$3120	$5800	$9285

Notes: [a] “—” represents an AE that was not experienced in any grade by ≥ 20% of patients treated with nivolumab plus ipilimumab in the CheckMate 214 trial, patients treated with nivolumab plus cabozantinib in the CheckMate 9ER trial, or not reported among patients treated with pembrolizumab plus lenvatinib in the KEYNOTE-581 trial per the KEYTRUDA prescribing information. [b] AE unit costs were based on AE-related hospitalization cost, which were estimated from the Healthcare Cost and Utilization Project (HCUP) 2017 National Inpatient Database based on the ICD-10 diagnosis codes associated with each AE. Cost inputs were inflated from 2017 USD to 2020 USD using an inflation factor of 1.0793 based on the Consumer Price Index (CPI) for all urban consumers in medical care service. [c] Total costs were calculated by multiplying each AE rate by the corresponding AE unit cost and reflect the rounded sum of the individual AE costs in each treatment arm.

Abbreviations: AE, adverse event; NIVO + CABO, nivolumab plus cabozantinib; NIVO + IPI, nivolumab plus ipilimumab; PEM + LEN, pembrolizumab plus lenvatinib; PPE, palmar-plantar erythrodysesthesia.

In each treatment arm, the top 5 AEs with the highest costs contributed to a majority of the total all-cause grade 3/4 AE cost. Hypertension, fatigue, weight loss, diarrhea, and hepatotoxicity accounted for 58% of the total all-cause grade 3/4 AE cost related to pembrolizumab plus lenvatinib. In the nivolumab plus cabozantinib arm, hypertension, hepatotoxicity, fatigue, diarrhea, and stomatitis accounted for 69% of the total all-cause grade 3/4 AE cost. For nivolumab plus ipilimumab, the main drivers were fatigue, hepatotoxicity, diarrhea, musculoskeletal disorders, and rash, which accounted for 83% of the total all-cause grade 3/4 AE cost (Table 3).

#### Treatment-Related Grade 3/4 AE Costs

The average treatment-related grade 3/4 AE costs for patients with aRCC who received nivolumab plus ipilimumab vs. nivolumab plus cabozantinib vs. pembrolizumab plus lenvatinib are presented in Fig. 1D and Table 4. Patients treated with pembrolizumab plus lenvatinib incurred treatment-related grade 3/4 AE costs of $5030 per patient; those who received nivolumab plus cabozantinib incurred $3162 in treatment-related grade 3/4 AE costs per patient (37% lower than pembrolizumab plus lenvatinib) while patients who received nivolumab plus ipilimumab incurred only $863 in costs per patient (83% lower than pembrolizumab plus lenvatinib).

**Table 4.** Treatment-related grade 3/4 AE costs associated nivolumab plus ipilimumab, nivolumab plus cabozantinib, and pembrolizumab plus lenvatinib (median follow-up of ~26.6 months).^a^

AE	Unit cost^b^	NIVO + IPI (*N* = 547)	NIVO + CABO (*N* = 320)	PEM + LEN (*N* = 352)
Asthenia	$13 679	—	—	$622
Decreased appetite	$9260	—	$116	$316
Diarrhea	$7736	$297	$508	$637
Dysgeusia	$7471	—	$0	—
Dysphonia	$6819	—	—	$0
Fatigue	$7979	$335	$199	$249
Hypertension	$7023	—	$812	$1756
Hypothyroidism	$9673	—	$30	$110
Increased ALT	$8504	—	$425	—
Increased AST	$8504	—	$292	—
Mucosal inflammation	$15 868	—	$149	—
Nausea	$7547	$110	$24	$129
PPE syndrome	$6480	—	$486	$258
Proteinuria	$7860	—	—	$581
Pruritus	$4690	$26	—	—
Rash	$6480	$95	$121	$221
Stomatitis	$8930	—	—	$152
Total cost^c^		$863	$3162	$5030

Notes: [a] “—” represents an AE that was not experienced in any grade by ≥ 20% of patients treated with nivolumab plus ipilimumab in the CheckMate 214 trial, nivolumab plus cabozantinib in the CheckMate 9ER trial or pembrolizumab plus lenvatinib in the KEYNOTE-426 trial, or not reported among patients treated with pembrolizumab plus axitinib in the KEYNOTE-581 trial, Motzer et al. [b] AE unit costs were based on AE-related hospitalization cost, which were estimated from the Healthcare Cost and Utilization Project (HCUP) 2017 National Inpatient Database based on the ICD-10 diagnosis codes associated with each AE. Cost inputs were inflated from 2017 USD to 2020 USD using an inflation factor of 1.0793 based on the Consumer Price Index (CPI) for all urban consumers in medical care service. [c] Total costs were calculated by multiplying each AE rate by the corresponding AE unit cost and reflect the rounded sum of the individual AE costs in each treatment arm.

Abbreviations: AE, adverse event; NIVO + CABO, nivolumab plus cabozantinib; NIVO + IPI, nivolumab plus ipilimumab; PEM + LEN, pembrolizumab plus lenvatinib; PPE, palmar-plantar erythrodysesthesia.

In each treatment arm, the top 5 AEs with the highest costs contributed to a majority or all of the total treatment-related grade 3/4 AE cost. Hypertension, diarrhea, asthenia, proteinuria, and decreased appetite accounted for 78% of the total treatment-related grade 3/4 AE cost associated with pembrolizumab plus lenvatinib. In the nivolumab plus cabozantinib arm, hypertension, diarrhea, PPE syndrome, increased ALT, and increased AST accounted for 80% of the total treatment-related grade 3/4 AE cost. For nivolumab plus ipilimumab, fatigue, diarrhea, nausea, rash, and pruritus accounted for 100% of the total treatment-related grade 3/4 AE cost (Table 4).

## Discussion

A solid understanding of the safety profile of available immunotherapy-based combinations is a crucial component of treatment optimization,^22,23^ allowing clinicians to maximize the benefit of novel first-line treatments for aRCC while properly managing the associated AEs and reducing ensuing costs. In the absence of head-to-head trials, the present study characterized the safety profiles of 4 novel immunotherapy-based combinations in patients with previously untreated aRCC and evaluated the costs associated with the all-cause and treatment-related grade 3/4 AEs. The nivolumab-based combinations were associated with lower average all-cause and treatment-related grade 3/4 AE costs per treated patient compared with the pembrolizumab-based combinations. The all-cause AE cost drivers (≥$500) of nivolumab-based combinations are fatigue/asthenia, hepatotoxicity, and diarrhea ($410 in the second comparison) for nivolumab plus ipilimumab, and hypertension, hepatotoxicity, fatigue/asthenia, diarrhea, and stomatitis/mucosal inflammation ($426 in the first comparison) for nivolumab plus cabozantinib. The all-cause AE cost drivers (≥$500) of pembrolizumab-based combinations are hepatotoxicity, hypertension, diarrhea, and fatigue/asthenia for pembrolizumab plus axitinib, and hypertension, fatigue, weight loss, diarrhea, hepatoxicity, and proteinuria for pembrolizumab plus lenvatinib.

The results show that the use of nivolumab-based combinations in first-line aRCC treatment leads to numerically lower grade 3/4 AE costs relative to pembrolizumab-based combinations. These findings align with the results of a prior cost-effectiveness analysis that compared the AE costs of nivolumab plus ipilimumab and pembrolizumab plus axitinib among patients from the all treated population in the intermediate/poor-risk subgroup, as defined by the International Metastatic Renal Cell Carcinoma Database Consortium.^24^ The AE cost estimate (based on grade 3 or higher AEs reported in ≥ 5.0% of patients of the respective pivotal trials) for nivolumab plus ipilimumab ($1151) was lower than the estimate for pembrolizumab plus axitinib ($3842). Together, the results provide valuable insight regarding the potential for cost savings associated with nivolumab-based combinations over pembrolizumab-based combinations, which can guide treatment decision making.

The top AE cost drivers varied across different immunotherapy-based combinations despite similarities. It is worth noting that some AEs such as fatigue, hypertension, and diarrhea, are known class effects of VEGF-targeted TKIs,^25-27^ Some others are often difficult to relate to any of the 2 classes of agents.^28-30^ Our results show that costs related to treatment-related hypertension and increased ALT and AST and diarrhea in the pembrolizumab plus axitinib arm drive the cost difference between this treatment arm and either of the nivolumab-based combinations. This may echo the findings of a recent network meta-analysis which showed that the combination of nivolumab plus ipilimumab was associated with lower rates of serious AEs than pembrolizumab plus axitinib.^31^ Given that severe AEs can lead to treatment discontinuation, additional health resource utilization, poorer clinical outcomes, and impact the quality of life in this vulnerable population,^32-34^ the different patterns of toxicities related to the use of immunotherapy-based combinations and the resulting clinical and economic impact for the patients, healthcare providers, and payers, must be considered when selecting the appropriate treatment strategy.

### Limitations

Results of this study should be considered along with certain limitations. First, the current analysis focused on costs associated with grade 3/4 AEs since these AEs are more prevalent, require medical treatment or hospitalization, and are more consistently reported in product prescribing information and the literature. Costs associated with grades 1, 2, and 5 AEs were not considered. As a result, the present analysis may slightly underestimate the total AE costs for the study cohorts. Further studies assessing the impact of grades 1, 2, and 5 AEs are warranted to supplement the findings from this analysis. Second, the unit costs for grade 3/4 AEs were consistently assumed to be the unit cost of one hospitalization with the corresponding diagnosis and were obtained from the 2017 US HCUP database. As some AEs, such as hypertension or PPE, may be less likely to require a hospitalization, the unit costs may not reflect the true costs incurred during the trial and may be subject to measurement errors which could result in either an underestimation or overestimation of costs. However, since this assumption was applied to all therapies, the impact on the comparative results between different therapies is limited. Third, the costs associated with other all-cause and treatment-related grade 3/4 AEs with corresponding any-grade AE rates less than 20% were not included in the analysis due to lack of such data for pembrolizumab plus axitinib in the KEYTRUDA prescribing information, although notably, nivolumab plus ipilimumab combination may be associated with rare but serious AEs, such as myocarditis,^35^ warranting further investigation. Fourth, indirect costs were, per definition, excluded from these analyses. Thus, further real-world studies are warranted to comprehensively assess these AE costs. Fifth, statistical significance testing was not conducted since aggregated trial-level data were used for pembrolizumab-based therapies. Finally, the study results were based on the AE profile of the patients in the CheckMate 214, CheckMate 9ER, KEYNOTE-426, and KEYNOTE-581 trials, and therefore, may not be generalizable to patients in real-world settings. Further studies with real-world data are warranted to compare the clinical and economic outcomes of these treatments.

## Conclusions

In this trial-based descriptive economic assessment of the all-cause and treatment-related AE costs of novel combination therapies for first-line treatment for patients with aRCC, the combinations of nivolumab plus ipilimumab and nivolumab plus cabozantinib were associated with lower grade 3/4 AE costs than pembrolizumab plus axitinib and pembrolizumab plus lenvatinib. For nivolumab plus ipilimumab, the fact that ipilimumab is stopped after the induction phase may contribute to lower observed AE costs. These results suggest that nivolumab-based combinations have a more favorable cost-benefit profile and offer clinicians and payers a therapeutic option for previously untreated aRCC that may reduce the substantial clinical and economic impacts in this population.

## Supplementary Material

oyac186_suppl_Supplementary_Tables

## Data Availability

The study has associated data in a data repository, which can be made available upon reasonable request to the corresponding author.
