# Supplementary material for: Grade 3/4 Adverse Event Costs of Immuno-oncology Combination Therapies for Previously Untreated Advanced Renal Cell Carcinoma
Source: Oncologist. 2023 Jan 18;28(1):72–9. doi: 10.1093/oncolo/oyac186 (PMC9847521; doi:10.1093/oncolo/oyac186)
Supplement: oyac186_suppl_Supplementary_Tables [file oyac186_suppl_supplementary_tables.docx]

**SUPPLEMENTAL MATERIAL**

**Supplemental Table 1. AE Categories in Prescribing Information for KEYTRUDA® (KEYNOTE-426) and the Corresponding AE in the IPD of the CheckMate 214 and CheckMate 9ER Trials^a,b^**

| **Prescribing Information for KEYTRUDA**® **(KEYNOTE-426)** | **IPD of the CheckMate 214 and CheckMate 9ER Trials** |
| --- | --- |
| **Diarrhea** | |
| Diarrhea | Diarrhea |
| Colitis | Colitis |
| Enterocolitis | Enterocolitis |
| Gastroenteritis | Gastroenteritis |
| Enteritis | Enteritis |
| Enterocolitis hemorrhagic | - |
| **Hypertension** | |
| Hypertension | Hypertension |
| Blood pressure increased | Blood pressure increased |
| Hypertensive crisis | Hypertensive crisis |
| Labile hypertension | - |
| **Hepatotoxicity** | |
| ALT increased | ALT increased |
| AST increased | AST increased |
| Autoimmune hepatitis | Autoimmune hepatitis |
| Blood bilirubin increased | Blood bilirubin increased |
| Hepatic enzyme increased | Hepatic enzyme increased |
| Hepatic function abnormal | Hepatic function abnormal |
| Hepatitis | Hepatitis |
| Hepatitis fulminant | Hepatitis acute |
| Immune-mediated hepatitis | - |
| Hepatocellular injury | Hepatocellular injury |
| Hepatotoxicity | Hepatotoxicity |
| Hyperbilirubinemia | Hyperbilirubinemia |
| Liver function test increased | Liver function test increased |
| Transaminases increased | Transaminases increased |
| Liver injury | - |
| Drug-induced liver injury | Drug-induced liver injury |
| **Rash** | |
| Rash | Rash |
| Rash generalized | Rash generalized |
| Perineal rash | - |
| Dermatitis | Dermatitis |
| Dermatitis atopic | Eczema |
| Seborrheic dermatitis | Seborrheic dermatitis |
| Dermatitis acneiform | Dermatitis acneiform |
| Dermatitis bullous | Dermatitis bullous |
| Dermatitis contact | Dermatitis contact |
| Exfoliative rash | Exfoliative rash |
| Genital rash | Genital rash |
| Rash erythematous | Rash erythematous |
| Rash macular | Rash macular |
| Rash maculo-papular | Rash maculo-papular |
| Rash papular | Rash papular |
| Rash pruritic | Rash pruritic |
| Skin exfoliation | Skin exfoliation |
| Skin discoloration | Skin discoloration |
| Butterfly rash | - |

**Abbreviations:**

*AE: adverse event; ALT: alanine aminotransferase; AST: aspartate aminotransferase; IPD: individual patient-level data; mFU: median follow-up.*

**Notes:**

[a] "-" represents AEs that were not available in the CheckMate 214 trial data (data cutoff: August 31, 2016) and CheckMate 9ER trial data (data cutoff: November 30, 2019).

[b] The list of AEs in the “Prescribing Information for KEYTRUDA® (KEYNOTE-426)” column was based on KEYTRUDA*®* prescribing information. The list of AEs in the “IPD of the CheckMate 214 and CheckMate 9ER Trials” column was obtained from IPD of CheckMate 214 trial or CheckMate 9ER trial.

**Supplemental Table 2. AE Categories in Prescribing Information for KEYTRUDA® (KEYNOTE-581) and the Corresponding AE in the IPD of the CheckMate 214 and CheckMate 9ER Trials^a-b^**

| **Prescribing Information for KEYTRUDA**®  **(KEYNOTE-581)** | **IPD of the CheckMate 214 and CheckMate 9ER Trials** |
| --- | --- |
| **Abdominal pain** | |
| Abdominal discomfort | Abdominal discomfort |
| Abdominal pain | Abdominal pain |
| Abdominal rigidity | Abdominal rigidity |
| Abdominal tenderness | Abdominal tenderness |
| Epigastric discomfort | Epigastric discomfort |
| Lower abdominal pain | Abdominal pain lower |
| Upper abdominal pain | Abdominal pain upper |
| **Acute kidney injury** | |
| Acute kidney injury | Acute kidney injury |
| Azotemia | Azotemia |
| Blood creatinine increased | Blood creatinine increased |
| Creatinine renal clearance decreased | - |
| Hypercreatininemia | Hypercreatininemia |
| Renal failure | Renal failure |
| Renal impairment | Renal impairment |
| Oliguria | Oliguria |
| Glomerular filtration rate decreased | Glomerular filtration rate decreased |
| Nephropathy toxic | Nephropathy toxic |
| **Decreased appetite** | |
| Decreased appetite | Decreased appetite |
| Early satiety | Early satiety |
| **Diarrhea** | |
| Diarrhea | Diarrhea |
| Gastroenteritis | Gastroenteritis |
| **Fatigue** | |
| Asthenia | Asthenia |
| Fatigue | Fatigue |
| Lethargy | Lethargy |
| Malaise | Malaise |
| **Hemorrhagic events** | |
| - | Hemorrhage |
| Anal hemorrhage | Anal hemorrhage |
| Aneurysm ruptured | - |
| Blood blister | Blood blister |
| Blood loss anemia | - |
| Blood urine present | Blood urine present |
| Catheter site hematoma | - |
| Cerebral microhemorrhage | Cerebral hemorrhage |
| Conjunctival hemorrhage | Conjunctival hemorrhage |
| Contusion | Contusion |
| Diarrhea hemorrhagic | Diarrhea hemorrhagic |
| Disseminated intravascular coagulation | Disseminated intravascular coagulation |
| Ecchymosis | Ecchymosis |
| Epistaxis | Epistaxis |
| Eye hemorrhage | Eye hemorrhage |
| Gastric hemorrhage | Gastric hemorrhage |
| Gastritis hemorrhagic | - |
| Gingival bleeding | Gingival bleeding |
| Hemorrhage urinary tract | - |
| Hemothorax | Hemothorax |
| Hematemesis | - |
| Hematoma | Hematoma |
| Hematochezia | Hematochezia |
| Hematuria | Hematuria |
| Hemoptysis | Hemoptysis |
| Hemorrhoidal hemorrhage | Hemorrhoidal hemorrhage |
| Increased tendency to bruise | Increased tendency to bruise |
| Injection site hematoma | - |
| Injection site hemorrhage | - |
| Intra-abdominal hemorrhage | Abdominal wall hematoma |
| Lower gastrointestinal hemorrhage | Lower gastrointestinal hemorrhage |
| Mallory-Weiss syndrome | Esophageal ulcer |
| Melaena | Melaena |
| Petechiae | Petechiae |
| Rectal hemorrhage | Rectal hemorrhage |
| Renal hemorrhage | Renal hemorrhage |
| Retroperitoneal hemorrhage | Retroperitoneal hemorrhage |
| Small intestinal hemorrhage | Small intestinal obstruction |
| Splinter hemorrhages | Splinter hemorrhages |
| Subcutaneous hematoma | Hemorrhage subcutaneous |
| Subdural hematoma | Hemorrhage intracranial |
| Subarachnoid hemorrhage | Subarachnoid hemorrhage |
| Thrombotic thrombocytopenic purpura | - |
| Tumor hemorrhage | Tumor hemorrhage |
| Traumatic hematoma | Traumatic hematoma |
| Upper gastrointestinal hemorrhage | Upper gastrointestinal hemorrhage |
| **Hepatotoxicity** | |
| ALT increased | ALT increased |
| AST increased | AST increased |
| Blood bilirubin increased | Blood bilirubin increased |
| Hepatic enzyme increased | Hepatic enzyme increased |
| Hepatic function abnormal | Hepatic function abnormal |
| Hepatic failure | Hepatic failure |
| Gamma-glutamyltransferase increased | Gamma-glutamyltransferase increased |
| Immune-mediated hepatitis | Autoimmune hepatitis |
| Hepatocellular injury | Hepatocellular injury |
| Hepatotoxicity | Hepatotoxicity |
| Hyperbilirubinemia | Hyperbilirubinemia |
| Hypertransaminasemia | Hypertransaminasemia |
| Liver function test increased | Liver function test increased |
| Transaminases increased | Transaminases increased |
| Liver injury | - |
| Drug-induced liver injury | Drug-induced liver injury |
| **Hypertension** | |
| Essential hypertension | Essential hypertension |
| Increased diastolic blood pressure | Diastolic hypertension |
| Hypertension | Hypertension |
| Increased blood pressure | Blood pressure increased |
| Hypertensive crisis | Hypertensive crisis |
| Hypertensive retinopathy | Retinopathy |
| Labile blood pressure | Blood pressure fluctuation |
| **Hypothyroidism** | |
| Hypothyroidism | Hypothyroidism |
| Increased blood thyroid stimulating hormone | Blood thyroid stimulating hormone increased |
| Secondary hypothyroidism | - |
| **Musculoskeletal disorders** | |
| Arthralgia | Arthralgia |
| Arthritis | Arthritis |
| Back pain | Back pain |
| Bone pain | Bone pain |
| Breast pain | Breast pain |
| Musculoskeletal chest pain | Musculoskeletal chest pain |
| Musculoskeletal discomfort | Musculoskeletal discomfort |
| Musculoskeletal pain | Musculoskeletal pain |
| Musculoskeletal stiffness | Musculoskeletal stiffness |
| Myalgia | Myalgia |
| Neck pain | Neck pain |
| Non-cardiac chest pain | Non-cardiac chest pain |
| Pain in extremity | Pain in extremity |
| Pain in jaw | Pain in jaw |
| **Palmar-plantar erythrodysesthesia syndrome** | |
| Palmar erythema | Palmar erythema |
| Palmar-plantar erythrodysesthesia syndrome | Palmar-plantar erythrodysesthesia syndrome |
| Plantar erythema | Plantar erythema |
| **Proteinuria** | |
| Hemoglobinuria | - |
| Nephrotic syndrome | Nephrotic syndrome |
| Proteinuria | Proteinuria |
| **Rash** | |
| Rash | Rash |
| Perineal rash | - |
| Rash pustular | Rash pustular |
| Genital rash | Genital rash |
| Infusion site rash | - |
| Penile rash | Penile rash |
| Rash erythematous | Rash erythematous |
| Rash macular | Rash macular |
| Rash maculo-papular | Rash maculo-papular |
| Rash papular | Rash papular |
| Rash pruritic | Rash pruritic |
| **Stomatitis** | |
| Aphthous ulcer | Aphthous ulcer |
| Gingival pain | Gingival pain |
| Glossitis | Glossitis |
| Glossodynia | Glossodynia |
| Mouth ulceration | Mouth ulceration |
| Mucosal inflammation | Mucosal inflammation |
| Oral discomfort | Oral discomfort |
| Oral mucosal blistering | Oral mucosal blistering |
| Oral pain | Oral pain |
| Oropharyngeal pain | Oropharyngeal pain |
| Pharyngeal inflammation | Pharyngeal inflammation |
| Stomatitis | Stomatitis |

**Abbreviations:**

*AE: adverse event; ALT: alanine aminotransferase; AST: aspartate aminotransferase; IPD: individual patient-level data; mFU: median follow-up.*

**Notes:**

[a] "-" represents AEs that were not available in the CheckMate 214 trial data (data cutoff: November 30, 2017) and CheckMate 9ER trial data (data cutoff: September 10, 2020) or reported in the KEYNOTE-581 trial data (data cutoff: August 28, 2020).

[b] The list of AEs in the “Prescribing Information for KEYTRUDA® (KEYNOTE-581)” column was based on KEYTRUDA*®* prescribing information. The list of AEs in the “IPD of the CheckMate 214 and CheckMate 9ER Trials” column was obtained from IPD of CheckMate 214 trial or CheckMate 9ER trial.
